# Exosome-Derived ADAM17 Promotes Liver Metastasis in Colorectal Cancer

**DOI:** 10.3389/fphar.2021.734351

**Published:** 2021-09-23

**Authors:** Jinbing Sun, Zhihua Lu, Wei Fu, Kuangyi Lu, Xiuwen Gu, Feng Xu, Jiamin Dai, Yang Yang, Jianlong Jiang

**Affiliations:** ^1^ Department of General Surgery, Changshu No. 1 People’s Hospital, Affiliated Changshu Hospital of Soochow University, Changshu, China; ^2^ Department of Radiology, Changshu No. 1 People’s Hospital, Affiliated Changshu Hospital of Soochow University, Changshu, China; ^3^ Department of Oncology, Changshu No. 1 People’s Hospital, Affiliated Changshu Hospital of Soochow University, Changshu, China; ^4^ Affiliated Hospital of Integrated Traditional Chinese and Western Medicine, Nanjing University of Chinese Medicine, Nanjing, China; ^5^ Department of Pharmacology, School of Pharmacy, Nanjing University of Chinese Medicine, Nanjing, China

**Keywords:** exosome, ADAM17, colorectal cancer, liver metastasis, E-cadherin

## Abstract

Exosomes derived from cancer cells are deemed important drivers of pre-metastatic niche formation at distant organs, but the underlying mechanisms of their effects remain largely unknow. Although the role of ADAM17 in cancer cells has been well studied, the secreted ADAM17 effects transported via exosomes are less understood. Herein, we show that the level of exosome-derived ADAM17 is elevated in the serum of patients with metastatic colorectal cancer as well as in metastatic colorectal cancer cells. Furthermore, exosomal ADAM17 was shown to promote the migratory ability of colorectal cancer cells by cleaving the E-cadherin junction. Moreover, exosomal ADAM17 overexpression as well as RNA interference results highlighted its function as a tumor metastasis-promoting factor in colorectal cancer *in vitro* and *in vivo*. Taken together, our current work suggests that exosomal ADAM17 is involved in pre-metastatic niche formation and may be utilized as a blood-based biomarker of colorectal cancer metastasis.

## Highlights

• Exosome-derived ADAM17 is elevated in the serum of patients with metastatic CRC.
• Exosomal ADAM17 is associated with the metastatic ability of CRC cell lines.
• CRC cell-secreted exosomal ADAM17 increases E-cadherin cleavage and cell migration.
• CRC cell-secreted exosomal ADAM17 promotes liver metastasis *in vivo*.

## 1 Introduction

Cancer metastasis is a multi-step, complex process during which tumor cells detach from the primary tumor site and finally seed in target distant organs where they proliferate to form metastatic nodules (Paul et al., 2017; Peinado et al., 2017). Several pre-metastatic niche biomarkers have been identified for cancer diagnosis and prognosis (Zhou et al., 2014; Zhang et al., 2017). Furthermore, targeting the pre-metastatic niche may represent a promising strategy for the prevention of cancer metastasis (Chafe et al., 2015; Zhang et al., 2015). Therefore, the identification of pre-metastatic niche formation-associated biomarkers for the diagnosis, prognosis, and prevention of cancer metastasis is of great relevance.

Exosomes are small vesicles ranging from 30 to 150 nm in size and contain proteins, lipids, and various nucleic acids (Liu et al., 2015). Furthermore, exosomes mediate signal transduction between neighboring and distant cells (Thery et al., 2002; Cocucci et al., 2009). These nano-sized vesicles have also been established as cancer-derived mediators that facilitate pre-metastatic niche formation in distant organs (Costa-Silva et al., 2015; Zhang and Wang, 2015; Becker et al., 2016; Liu et al., 2016). Recently, tumor-secreted exosomes were reported to facilitate cancer-induced vascular permeability and inflammation (Costa-Silva et al., 2015; Hoshino et al., 2015). Importantly, exosomes obtained from the serum of patients with cancer have proven to be reliable markers for cancer diagnosis (Wang et al., 2017; Zhang et al., 2017; Rodrigues et al., 2019). While exosomes are known carriers of miRNAs, mRNA, and long non-coding RNA (Li et al., 2013; Bollati et al., 2015; Chen et al., 2020), the role of exosomal protein, especially membrane-bound proteins, is not yet fully understood. A disintegrin and metalloproteinase 17 (ADAM17), also known as tumor necrosis factor-alpha converting enzyme, is a membrane protein of the ADAM protein family (Scheller et al., 2011). ADAM17 was found to be highly expressed in various types of tumors as well as to affect tumor progression (Zhang et al., 2012). Our previous study revealed that ADAM17 overexpression in digestive tract malignancies was closely associated with tumor proliferation and metastasis (Sun et al., 2017). However, the precise molecular mechanism underlying the role of cancer cell-derived exosomal ADAM17 in metastasis remains unclear.

Herein, we identified that colorectal cancer (CRC) cell-derived exosomal ADAM17 is strongly associated with metastasis in patients with CRC as well as with the metastatic ability of CRC cells. Furthermore, exosomal ADAM17 was shown to effectively enhance the migratory ability of CRC cells by cleaving E-cadherin junctions. Exosomal ADAM17 upregulation and RNA interference *in vivo* and *in vitro* confirmed the function of exosomal ADAM17 as a tumor metastasis-promoting factor in CRC. Further, our clinical data suggested that circulating exosome-derived ADAM17 may be utilized as a blood-based biomarker for the prediction of metastasis in patients with CRC.

## 2 Materials and Methods

### 2.1 Reagents

GW4869 was obtained from Sigma-Aldrich (St. Louis, MO, United States). Lipofectamine® 3,000 Transfection Reagent was purchased from Thermo Scientific (USA). RIPA lysis buffer was purchased from Beyotime (Jiangsu, China). Primary antibodies for immunoblot analysis included anti-ADAM17 (ab39163), anti-CD81 (ab79559), anti-E-cadherin (ab231303), anti-N-cadherin (ab76011), anti-vimentin (ab92547), anti-snail (ab180714), and anti-β-actin (ab8226) (Abcam, Cambridge, MA, United States). Goat anti-rabbit IgG and goat anti-mouse IgG antibodies were purchased from LI-COR (Lincoln, NE, United States).

### 2.2 Specimen Collection

Human peripheral blood samples from 20 patients with CRC, with and without liver metastasis, were obtained from the Department of General Surgery of the Changshu No. 1 People’s Hospital affiliated to Soochow University. The patients underwent surgical resection in our department of gastrointestinal surgery between 2015 and 2016. The International Union Against Cancer (UICC)/American Joint Committee on Cancer (AJCC) TNM staging modified in 2003 was applied for colorectal cancer staging. The two groups of patients were matched by age and sex, and all cases were pathologically confirmed (Supplementary Table S1). Informed consent was provided from all individuals for blood donation using approved institutional protocols. Tubes containing EDTA were applied to collect blood samples and centrifuged at 2,500×g for 10 min to extract the serum for further study (Zeng et al., 2018). Plasma exosomes were isolated as described in Section 2.5 below.

All procedures performed in studies involving human participants were in accordance with the ethical standards of the institutional and/or national research committee as well as the 1964 Helsinki declaration and its later amendments or comparable ethical standards. This study was approved by the Ethics Committee of Changshu No. 1 People’s Hospital Affiliated to Soochow University. Informed consent was obtained from all participants included in the study (Sun et al., 2017).

### 2.3 Animal Models

Six-week-old male athymic BALB/c-nu/nu mice were purchased from the Beijing Vital River Laboratory Animal Technology Co., Ltd. and maintained in a specific pathogen-free environment. All protocols for animal studies were reviewed and approved by the Institutional Animal Care and Use Committee of Changshu No. 1 People’s Hospital Affiliated to Soochow University. For the orthotropic metastasis assay, nude mice were anesthetized, and their ceca were exteriorized by laparotomy. Thereafter, 2×10^6^ CRC cells were injected into the mesentery at the tail end of the cecum. To analyze the role of exosomes in tumor metastasis, 10 µg of CRC-derived exosomes were intravenously injected every 3 days after the implantation of CRC cells. After 60 days, the mice were sacrificed, and liver metastases were quantified. To evaluate metastasis, serial sections from the livers were stained with hematoxylin-eosin (HE) and screened for metastatic nodules.

### 2.4 Cell Culture

Human colorectal carcinoma SW480, SW620, Lovo, DLD-1, and HCT-116 cell lines were purchased from the Cell Bank of the Shanghai Institute of Biochemistry and Cell Biology (China), and human colonic epithelial cells (HCoEpiC) were purchased from ScienCell (USA). Cells were cultured in DMEM (for HCT-116 cells), Ham’s F-12K (Kaighn’s) medium (for Lovo cells), RPMI-1640 (for DLD-1 cells), L-15 medium (for SW620 and SW480 cells), and colonic epithelial cell medium (for HCoEpiC), supplemented with 10% fetal bovine serum (FBS, except for HCoEpiC), 100 U/mL penicillin, and 100 μg/ml streptomycin (all available from Invitrogen, Grand Island, NY, United States). The FBS used for conditioned medium (CM) collection, exosome isolation, and endothelial cell treatment was depleted of exosomes via overnight centrifugation at 100,000×g and 4°C. All cell lines were cultured under a humidified atmosphere in a 5% CO_2_ incubator at 37°C.

### 2.5 Exosome Isolation, Characterization, and Analyses

Exosomes were purified from CRC cell culture CM or CRC patient serum via ultracentrifugation according to a previous exosome extraction method (Zeng et al., 2018). The amount of exosomal protein was determined using the BCA Protein Assay kit (Beyotime Biotechnology, China). For transmission electron microscopy analysis, exosomes were fixed with 2% paraformaldehyde and observed using a transmission electron microscope (Hitachi H-7500, Japan) (Zeng et al., 2018). The number and size of exosomes were directly tracked using the Nanosight NS 300 system (NanoSight Technology, Malvern, United Kingdom), and data were analyzed with the NTA analytical software (version 2.3).

### 2.6 Western Blot Analysis and Quantitative Proteomic Analysis

For western blot analysis, total protein was extracted from exosomes, CRC cells, or mouse liver metastasis tissue using RIPA lysis buffer and analyzed as previously described (Fang et al., 2020). The exosomes were labeled with iTRAQ reagents using the iTRAQ multiplex kit (AB Sciex, USA) and followed analyzed according to previous publication (Kawakami et al., 2017). Labeled samples were separated and automatically spotted onto a MALDI plate, and mass spectra were acquired using the AB Sciex TOF/TOF 5800 system. All MS/MS data were analyzed via MASCOT and the Protein Pilot software (version 4.5; AB Sciex) to identify and quantify corresponding proteins in different groups (Supplementary Table S2). Protein identification was considered correct, based on the selection criteria (Kawakami et al., 2017).

### 2.7 DNA Constructs and RNA Interference Studies

Inhibition of ADAM17 expression in CRC cell-derived exosomes was performed using ADAM17-directed siRNAs. Human ADAM17-specific siRNA 5′-TGAGGCAG TCT​CTC​CTA​TTC​CTG​ACC​AGC-3′ and nonsense siRNA: 5′-TGA​CCA​CCC​TGA​CCT​ACG​GCG​TGC​AGT​GC-3′ were obtained from RiboBio (Guangzhou, China). The pcDNA3.1-ADAM17 plasmid was constructed using standard techniques. Briefly, DNA fragments encoding ADAM17 were generated via high-fidelity PCR and cloned into the pcDNA3.1 vector. All constructs were confirmed via DNA sequencing and purified using an Endofree Plasmid Preparation Kit (QIAGEN, Valencia, CA, United States). Cells were transfected with pcDNA3.1, pcDNA3.1-ADAM17, ADAM17-siRNA, and nonsense (scrambled) control siRNA (NC-siRNA) using a liposome-based method for 48 h, and the cells were harvested for subsequent analyses.

### 2.8 Wound-Healing Assay and Migration Assay

Cell wound-healing and migration assays were performed as previously described (Wang et al., 2015; Xu et al., 2016).

### 2.9 Statistical Analysis

The experimental data were analyzed using the Student’s t-test and one-way analysis of variance (ANOVA) to determine significant differences between the two groups. By applying a Chi-square test, the relationship between the CirExo-ADAM17 and clinicopathological features (such as gender, age, TNM stage, metastasis, etc.) in colorectal cancer patients who underwent surgical excision were then compared. The Kaplan-Meier survival curve was used to evaluate the relationship between CirExo-ADAM17 and survival outcomes in different patients with colorectal cancer. Significance tests used log-rank test analysis. Univariate survival analysis was further conducted to evaluate the effect of CirExo-ADAM17 on the prognosis of colorectal cancer. Data are presented as the mean ± SD. Western blotting analyses were repeated three times, and the results were quantified using the ImageJ software. Statistical analysis was performed using the SPSS 13.0 software (SPSS Inc., Chicago, IL, United States). Statistical significance was set at *p* < 0.05.

## 3 Results

### 3.1 Exosomal ADAM17 is Upregulated in Metastatic Compared to Non-metastatic CRC

**FIGURE 1:**
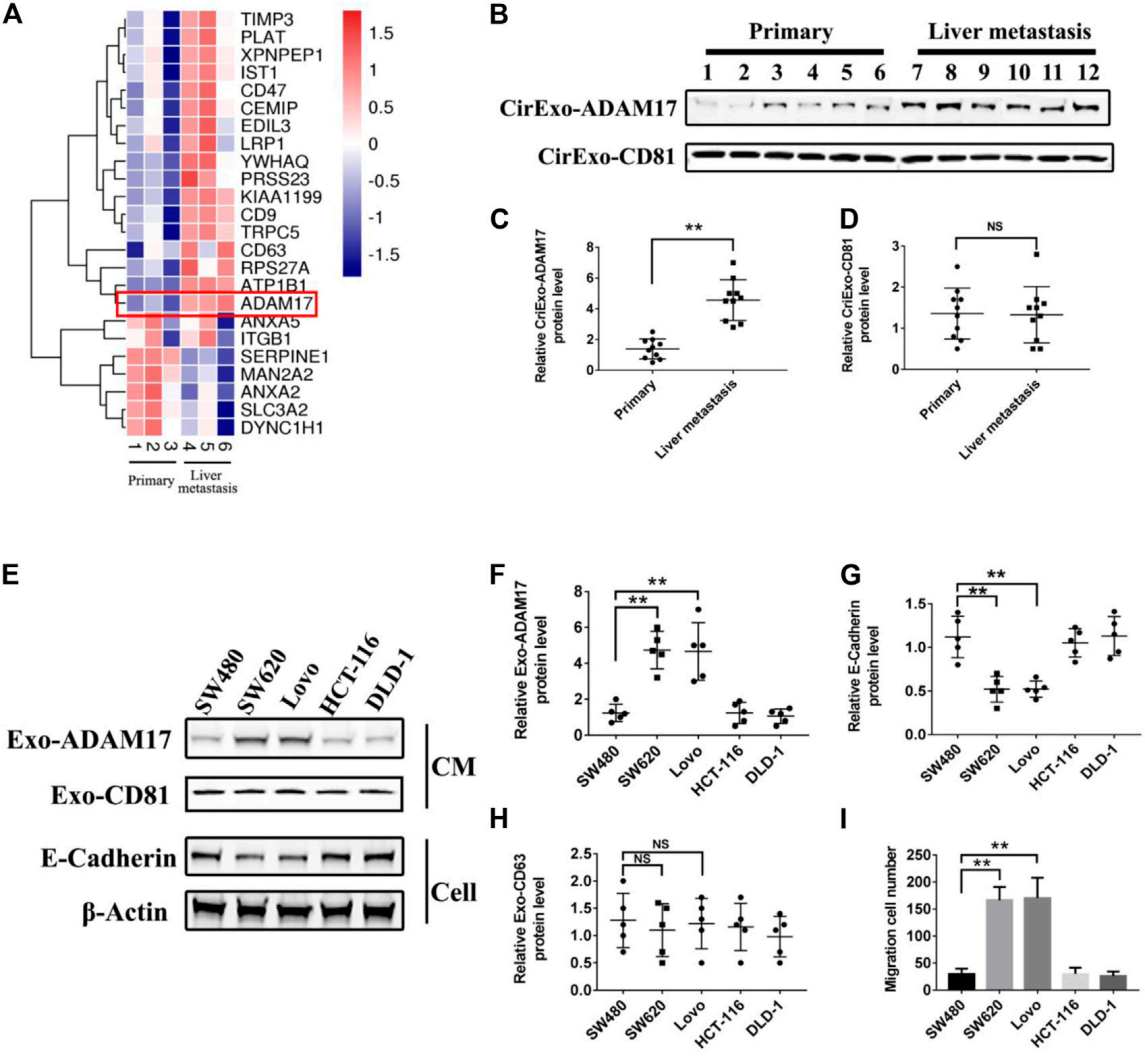
ADAM17 levels in exosomes from CRC patient sera and CRC cell lines. **(A)** Proteomic analysis of serum-derived exosomal protein differentially expressed between non-metastatic (primary) and liver metastastatic patients with CRC. A heatmap of differentially expressed exosomal proteins based on quantitative mass spectrometry values (technical triplicates, * false discovery rate < 0.05 *via* ANOVA). Hierarchical clustering (1—the sample Spearman’s rank correlation coefficient between observations) was performed on protein expression levels (*n* = 3). Exosomal ADAM17 was marked with a red frame as a differentially expressed protein. **(B)** The serum-derived exosomes of non-metastatic (primary) and liver metastatic patients with CRC were collected and lysed for analysis of ADAM17 and CD81 protein levels. **(C,D)** The relative protein levels were further calculated via gray analysis (the total exosomal protein was set as the internal control, *n* = 10). **(E)** SW480, SW620, Lovo, HCT-116, and DLD-1 exosomes and cells were subjected to analysis of ADAM17, CD81, and E-cadherin protein levels (CM, conditioned medium). **(F–H)** The relative protein levels were further calculated via gray analysis (the total exosomal protein was set as the internal control for exosomal protein analysis, *ß*-actin was set as the internal control for cells, *n* = 6). **(I)** Cell migration assessment of SW480, SW620, Lovo, HCT-116, and DLD-1 cells *via* transwell assays (*n* = 6). Data are expressed as means ± SDs. *, *p* < 0.05; **, *p* < 0.01.

Studies have shown that tumor-derived exosomes package specific proteins critical for metastasis into target organs (Peinado et al., 2012; Costa-Silva et al., 2015). Quantitative mass spectrometry comparison of exosomal proteomes revealed that only 24 proteins were differentially expressed in exosomes from liver metastases of patients with CRC when compared to exosomes from patients without metastasis (Figure 1A; Supplementary Table S2). Combining the protein function in tumorigenesis and development, ADAM17 emerged as a prominent exosomal protein in liver metastasis-derived exosomes, with a low expression in exosomes from patients without metastasis and from healthy donors, suggestive of its specific association with CRC liver metastasis potential (Figures 1A–D; Supplementary Figure S1A). Moreover, it was observed that CirExo-ADAM17 had a significant impact on the postoperative prognosis of patients with colorectal cancer. Kaplan-Meier univariate survival analysis results indicated that CirExo-ADAM17 (*p* < 0.05) was associated with the poor postoperative prognosis (Supplementary Tables S1, S3, and Supplementary Figure S2). Furthermore, exosomal ADAM17 levels were significantly increased in metastatic CRC cell lines (SW620 and Lovo) relative to those in SW480, DLD-1, HCT 116, and human colonic epithelial cells (HCoEpiC), as determined via western blot analysis (Figures 1E,F,H; Supplementary Figure S1B). Lower E-cadherin protein levels were detected in SW620 and Lovo cells, in agreement with their high migratory ability (Figures 1E,G,I). Taken together, our data identified ADAM17 as a protein upregulated in exosomes from metastatic CRC cells.

### 3.2 Exosomal ADAM17 Enhanced the Migratory Properties of CRC Cells

**FIGURE 2:**
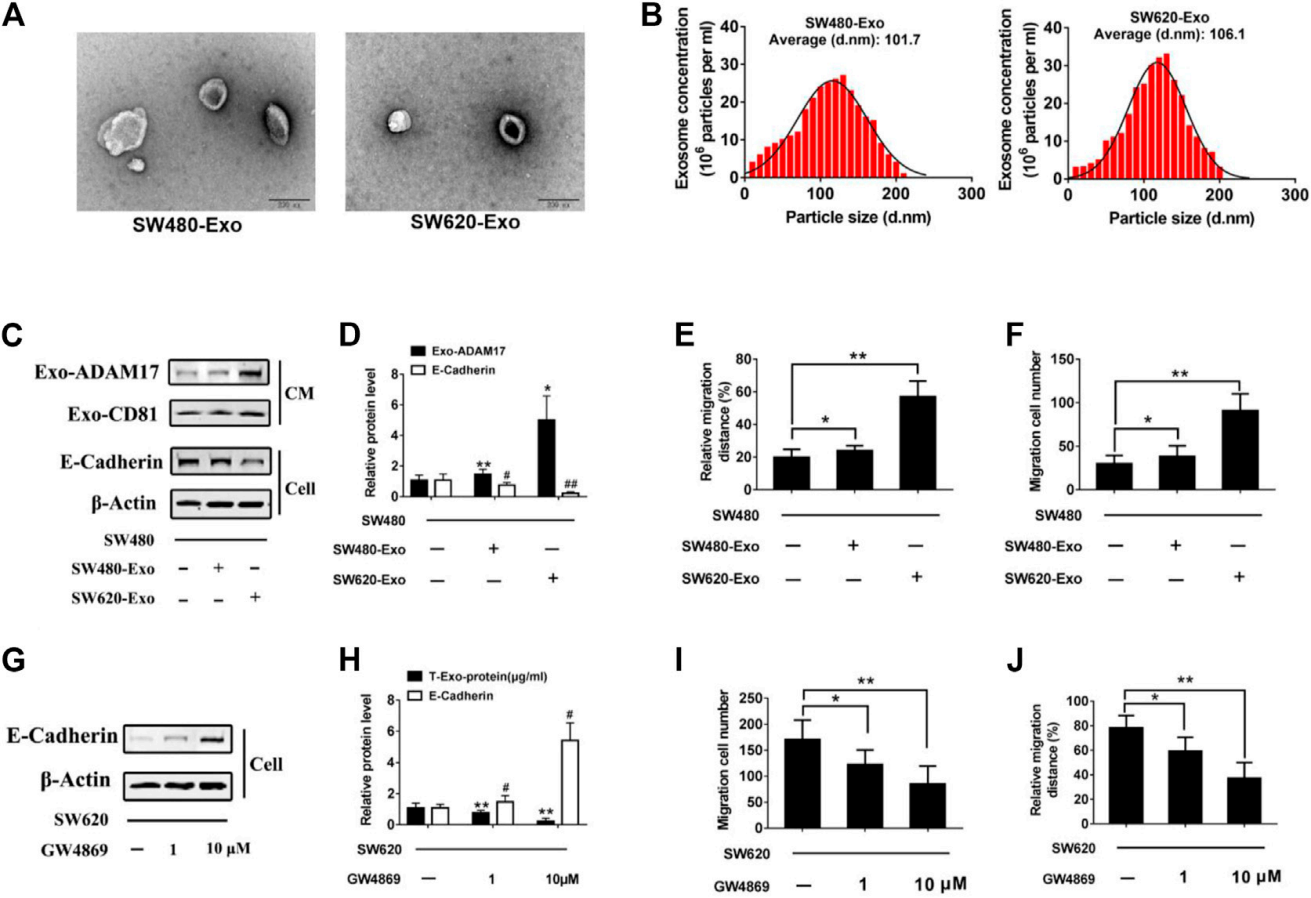
Exosomal ADAM17 enhances the migratory properties of CRC cells. Characterization of CRC cell-derived exosomes. **(A)** Electron microscope images of exosomes isolated from the CM of cultured SW480 and SW620 cells. Scale bar, 200 nm. **(B)** Nanoparticle tracking analysis of isolated exosomes. **(C,D)** SW480 cells were treated with SW480-derived exosomes (SW480-Exo) and SW620-derived exosomes (SW620-Exo) at 50 µg (10 μg/ml) per 1×10^5^ cultured cells. Exosomes were collected, cells were lysed, and protein levels were further calculated via gray analysis (the total exosomal protein was set as the internal control for exosomal protein analysis, *ß*-actin was set as the internal control for cells, *n* = 6). **(E,F)** Changes in migration were measured via transwell and wound scratch assays using SW480 cells treated with SW480-Exo and SW620-Exo (*n* = 6). **(G,H)** SW620 cells were treated with different concentration GW4869, and the total exosomal protein as well as cellular E-cadherin levels were calculated (*n* = 6). **(I,J)** Changes in migration were measured via transwell and wound scratch assays using SW620 cells treated with GW4869 (*n* = 6). Data are expressed as means ± SDs. * or #, *p* < 0.05; ** or ##, *p* < 0.01.

To study the role of exosomes in CRC metastasis, we isolated exosomes from two colorectal cancer cell lines with different migratory ability, SW480 and SW620. SW480- and SW620-derived exosomes were 107.2 nm (SW480-Exo) and 115.1 nm (SW620-Exo) in size (Figures 2A,B). SW620-Exo, which had higher ADAM17 levels, stimulated the migration of SW480 cells, reducing E-cadherin protein levels relative to those in SW480-Exo-treated cells (Figures 2C–F). To further identify the role of exosomes in the SW620-mediated promotion of migration, the exosome biogenesis/release inhibitor GW4869 (Essandoh et al., 2015) was applied to SW620 cells. GW4869 decreased SW620 exosome production in a dose-dependent manner and further decreased the migratory properties of SW620 cells (Figures 2G–J). These results demonstrated that CRC-derived exosomes harbored the ADAM17 oncoprotein, which may play an important role in the exosome-mediated stimulation of CRC metastasis.

### 3.3 Exosomal ADAM17 Increased the Cleavage of E-Cadherin

**FIGURE 3:**
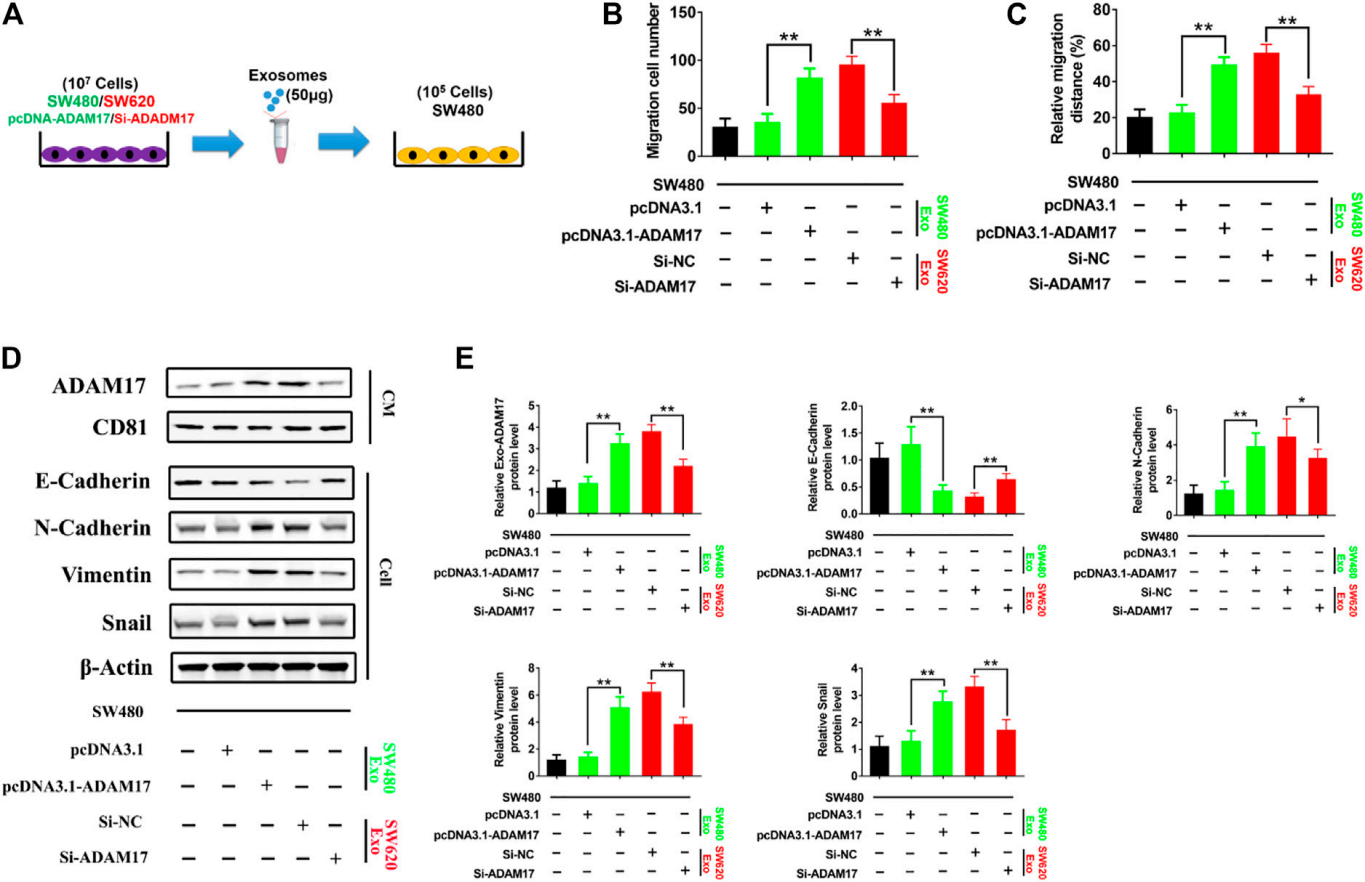
Exosomal ADAM17 enhances the cleavage of E-cadherin. **(A)** Schematic description of the experimental design. The pcDNA3.1-, pcDNA3.1-ADAM17-transfected SW480-derived exosomes and Si-NC-, Si-ADAM17-transfected SW620-derived exosomes were isolated. 50 μg of exosomes were added to 5×10^5^ SW480 cells. **(B,C)** Changes in migration were measured via transwell and wound scratch assays using SW480 treated with pcDNA3.1-, pcDNA3.1-ADAM17-transfected SW480-derived exosomes as well as Si-NC-, Si-ADAM17-transfected SW620-derived exosomes (*n* = 6). **(D,E)** SW480 cells were treated with pcDNA3.1-, pcDNA3.1-ADAM17-transfected SW480-derived exosomes and Si-NC-, Si-ADAM17-transfected SW620-derived exosomes. Exosomes and lysed cells were collected for the assessment of protein levels via gray analysis (the total exosomal protein was set as the internal control for exosomal protein analysis, *ß*-actin was set as the internal control for cells, *n* = 6). Data are expressed as means ± SDs. *, *p* < 0.05; **, *p* < 0.01.

To further explore the role of exosomal ADAM17 in CRC metastasis, ADAM17-overexpressing SW480-Exo and ADAM17-knockdown SW620-Exo were applied to SW480 cells (Figures 3A,D,E). SW480-Exo treatment enhanced the migratory properties of SW480 cells, whereas ADAM17 knockdown in SW620-Exo eliminated their migration-stimulating effect on SW480 cells (Figures 3B,C). E-cadherin prevents cancer cell dissemination from the primary lesion to distant organs by decreasing motility, migratory, and invasive properties (Gogali et al., 2010). E-cadherin is an important substrate of ADAM17. Its shedding requires the cleavage of α-secretase in the extracellular membrane, which is catalyzed by ADAM17 (Ma et al., 2020). E-cadherin levels were decreased following treatment with ADAM17-overexpressing SW480-Exo and increased after treatment with ADAM17-knockdown SW620-Exo (Figures 3D,E). Epithelial-mesenchymal transition (EMT) plays a critical role in cancer progression and metastasis (Huang et al., 2015), during which considerable morphological transformation occurs, in parallel to the suppression of epithelial markers (Zheng and Kang, 2014). The relative protein levels of N-cadherin, vimentin, and Snail were increased after treatment with ADAM17-overexpressing SW480-Exo and decreased following treatment with the ADAM17-knockdown SW620-Exo (Figures 3D,E). Taken together, CRC-derived exosomal ADAM17 stimulated cancer cell migration via cleavage of E-cadherin and the upregulation mesenchymal expression.

### 3.4 Colorectal Cancer Cell-Secreted Exosomal ADAM17 Promotes Metastasis *In vivo*

**FIGURE 4:**
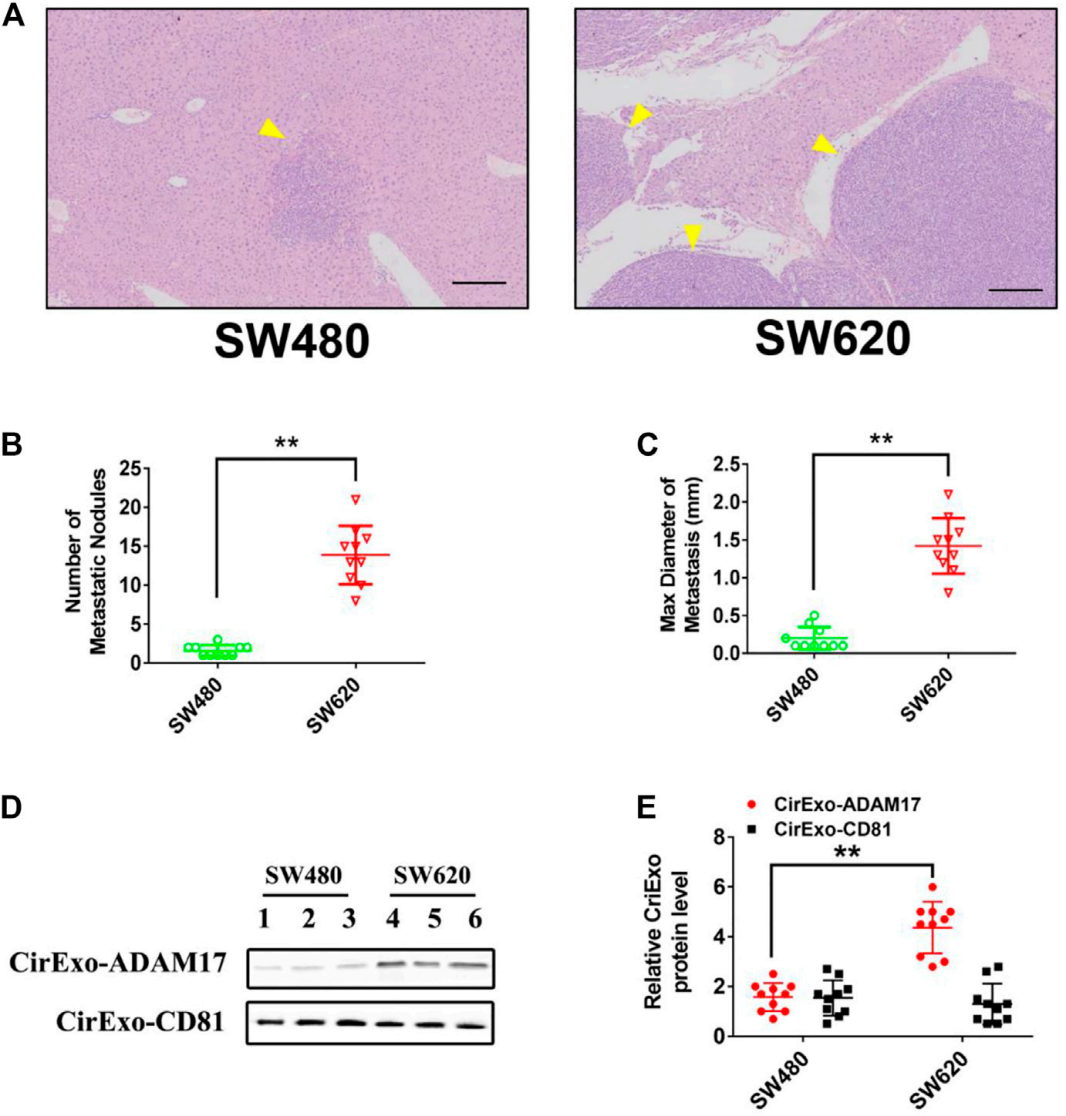
Exosomes derived from CRC cells promote liver metastasis. SW480 and SW620 cells were implanted into the mesentery at the tail end of the cecum. Autopsies were performed, and the presence of metastases was examined macroscopically 60 days after CRC implantation. **(A)** Representative H&E staining results of liver slices from SW480- and SW620-implanted mice. Arrows indicate the tumor nodules. Scale bar, 250 μm. **(B,C)** The number and maximal diameter of metastatic nodules in the livers of SW480- and SW620-implanted mice were calculated and analyzed (*n* = 10). **(D,E)** The serum-derived exosomes of SW480- and SW620-implanted mice were collected and lysed for analysis of ADAM17 and CD81 protein levels. The relative protein levels were further calculated via gray analysis (the total exosomal protein was set as the internal control, *n* = 10). Data are expressed as means ± SDs. *, *p* < 0.05; **, *p* < 0.01.

**FIGURE 5:**
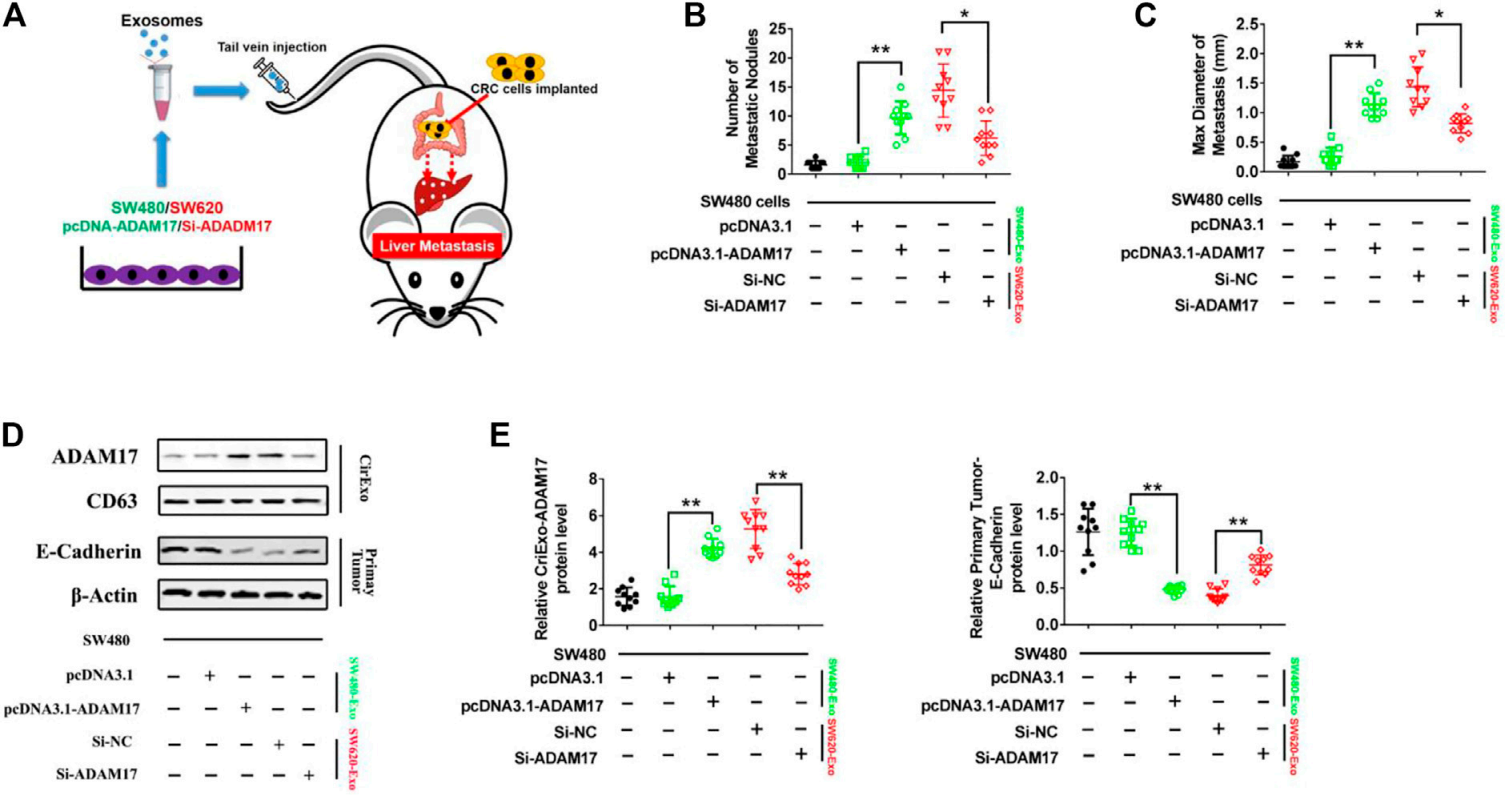
CRC cell-secreted exosomal ADAM17 promotes metastasis *in vivo* ***.* (A)** Schematic description of the experimental design. The pcDNA3.1-, pcDNA3.1-ADAM17-transfected SW480-derived exosomes and Si-NC-, Si-ADAM17-transfected SW620-derived exosomes were isolated. 10 μg exosomes were used to intravenously inject mice every 3 days for 2 months after the implantation of SW480 cells. **(B,C)** The number and maximal diameter of metastatic nodules in the livers of SW480-implanted mice treated with pcDNA3.1-, pcDNA3.1-ADAM17-transfected SW480-derived exosomes and Si-NC-, Si-ADAM17-transfected SW620-derived exosomes were calculated and analyzed (*n* = 10). **(D,E)** The serum-derived exosomes and primary tumors of SW480-implanted mice treated with pcDNA3.1-, pcDNA3.1-ADAM17-transfected SW480-derived exosomes and Si-NC-, Si-ADAM17-transfected SW620-derived exosomes were collected and lysed for the assessment of ADAM17, CD81, and E-cadherin protein levels. The relative protein levels were determined via gray analysis (the total exosomal protein was set as the internal control for exosomal protein analysis, *ß*-actin was set as the internal control for cells, *n* = 6). Data are expressed as means ± SDs. *, *p* < 0.05; **, *p* < 0.01.

To determine whether CRC-derived exosomes play a role in liver metastasis, we established a mouse CRC tumor model. SW480 and SW620 cells were implanted into nude mice separately. The SW620 implanted mice had higher rates of hepatic metastases, and also showed more and larger metastatic nodules in the liver compared with those in the SW480 implanted mice (Figures 4A,B). In addition, serum-derived exosomes were isolated, and higher exosomal ADAM17 protein levels were found in SW620 implanted mice compared to those in SW480 (Figures 4C,D). Further, to identify the role of exosomal ADAM17 in CRC metastasis, ADAM17 overexpression in SW480-Exo and ADAM17 downregulation in SW620-Exo were applied to SW480 cells implanted in mice (Figures 5A,D,E). ADAM17 overexpression in SW480-Exo increased the hepatic metastases rates and showed more and larger metastatic nodules in SW480 cells implanted in the mouse liver, whereas ADAM17 downregulation in SW620-Exo eliminated the SW620-Exo liver metastasis stimulation ability in SW480 cells (Figures 5B,C). Furthermore, the E-cadherin level was decreased in ADAM17 overexpression in the SW480-Exo group and increased in ADAM17 downregulation in the SW620-Exo group (Figures 5D,E). These findings strongly indicate that colorectal cancer cell-secreted exosomal ADAM17 promotes metastasis *in vivo*.

## 4 Discussion

CRC-derived exosomes are expected to play a role in the generation of metastatic microenvironments (Shao et al., 2018; Lafitte et al., 2019; Lin et al., 2019), but the underlying molecular mechanisms have not yet been fully determined. This study elucidated the effect of exosomal ADAM17 derived from SW620 cells, a highly liver metastatic CRC cell line, on the promotion of liver metastasis with poorly metastatic SW480 cells. Studies on lung cancer and melanoma have revealed that exosomes are involved in the regulation of angiogenesis and the EMT (Grange et al., 2011). However, as the role of exosomes in cancer metastasis might vary between tumor types, the underlying mechanisms through which exosomes regulate CRC liver metastasis should be elucidated (Wang et al., 2015).

The sheddase enzymes on the exosomal surface have rarely been studied. As a sheddase, ADAM17 cleaves the extracellular domains of transmembrane proteins, thereby releasing them and modulating cell–cell and cell–environment communication (Dusterhoft et al., 2019). ADAM17 plays a profound role in colonic tumorigenesis, and its expression was shown to be increased in tissue samples from patients with CRC (Walkiewicz et al., 2016). As an oncogene, the role of exosome-derived ADAM17 in CRC metastasis remains to be elucidated. Our study identified ADAM17 as a robust serum marker in metastatic CRC patients, in addition to its upregulation in metastatic CRC cells. As a key adhesive molecule in the prevention of tumor progression, E-cadherin is processed via proteolytic modifications (Puisieux et al., 2014; Ma et al., 2020). In particular, E-cadherin shedding requires α-secretase cleavage, which is catalyzed by ADAM17 (Ma et al., 2020). Our study revealed that exosomal ADAM17 effectively enhanced the migratory ability of CRC cells via E-cadherin cleavage. EMT is a central cellular process in cancer metastasis, characterized by the loss of cell–cell junctions and a decrease in the expression of the epithelial marker E-cadherin, in parallel with the upregulation of the mesenchymal marker N-cadherin and cytoskeleton rearrangements intended for enhanced invasive capacity (Tennakoon et al., 2015). Exosomal ADAM17 increased the expression of mesenchymal markers, such as N-cadherin, vimentin, and Snail, in turn promoting EMT in CRC. To verify the direct role of exosomes in CRC metastasis *in vivo*, we established liver metastatic CRC nude mouse models through the implantation of SW620 and SW480 cells, followed by an assessment of liver metastasis frequency and severity. The SW620-implanted mice had higher rates of hepatic metastases, accompanied by high serum exosomal ADAM17 protein levels. Our study further confirmed the role of exosomal ADAM17 in CRC metastasis via ADAM17 overexpression and knockdown in related CRC cell-derived exosomes. The E-cadherin level in primary CRC tumors was decreased following treatment with ADAM17-overexpressing exosomes, in parallel with an increased rate of hepatic metastasis, as well as more and larger metastatic nodules in the livers of CRC cell-implanted mice. These results highlighted the importance of exosomal ADAM17 in CRC liver metastasis.

**FIGURE 6:**
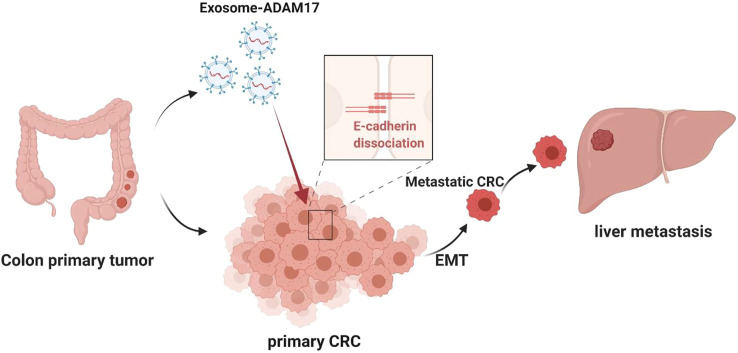
Proposed mechanism for exosomal ADAM17-mediated promotion of CRC metastasis. The CRC-derived exosomal ADAM17 promoted the E-cadherin cleavage, enhancing the migratory properties of CRC cells, which in turn promoted hepatic metastasis *in vivo*.

In the present study, we demonstrated the pivotal role of exosomal ADAM17 in CRC liver metastasis, suggesting that it might function through the regulation of cellular migration, thus highlighting its potential as a prognostic biomarker and therapeutic target. We also demonstrated that the effects of exosomal ADAM17 are mediated via E-cadherin cleavage, which promotes cancer cell dissemination from the primary lesion to distant organs by enhancing motility as well as migratory and invasive properties (Figure 6). Our research furthers the understanding of how exosomes regulate CRC liver metastasis and provides a basis for the development of associated therapeutics. Future studies should focus on confirming tumor-derived exosomal ADAM17 as a plasma-based biomarker for the non-invasive screening of patients with CRC, as well as on the assessment of targeted ADAM17-based therapeutics against CRC liver metastasis.

## Data Availability

The original contributions presented in the study are publicly available. This data can be found here: A list of SWISS MODEL accessions can be found in the Supplementary Material.

## Abbreviations

ADMA17, a disintegrin and metalloprotease 17; CM, conditioned medium; CRC, colorectal cancer; HCoEpiC, human colonic epithelial cells; PBS, phosphate-buffered saline; EMT, epithelial-mesenchymal transition.
