# Supplementary material for: Exosome-Derived ADAM17 Promotes Liver Metastasis in Colorectal Cancer
Source: Front Pharmacol. 2021 Sep 23;12:734351. doi: 10.3389/fphar.2021.734351 (PMC8506248; doi:10.3389/fphar.2021.734351)
Supplement: Supplementary file 1 [file DataSheet1.docx]

**Table S1: Relationship between CirExo-ADAM17 protein expression and the clinicopathological characteristics in colorectal carcinoma**

| Clinical characteristics | Cases, *n* | Positive CirExo-ADAM17 expression, *n* (%) | P value | χ2 |
| --- | --- | --- | --- | --- |
| Age |  |  |  |  |
| ≥60 years | 11 | 6 (54.55) | 0.964 | 0.002 |
| <60 years | 9 | 5 (55.56) |  |  |
| Sex |  |  |  |  |
| Male | 10 | 5 (50.00) | 0.653 | 0.202 |
| Female | 10 | 6 (60.00) |  |  |
| Histology |  |  |  |  |
| Adenocarcinoma | 12 | 6 (50.00) | 0.582 | 0.303 |
| Mucinous | 8 | 5 (62.50) |  |  |
| CEA |  |  |  |  |
| < 200 ng/ml | 11 | 6 (54.55) | 0.964 | 0.002 |
| > 200 ng/ml | 9 | 5 (55.56) |  |  |
| Distant metastasis |  |  |  |  |
| M0 | 10 | 2 (20.00) | 0.001 | 9.899 |
| M1 | 10 | 9 (90.00) |  |  |
| KRAS status |  |  |  |  |
| Mutant | 11 | 6 (54.55) | 0.964 | 0.002 |
| Wild type | 9 | 5 (55.56) |  |  |
| TNM staging |  |  |  |  |
| Phase I | 10 | 3 (30.00) | 0.024 | 5.051 |
| Phase II and III | 10 | 8 (80.00) |  |  |

**Table S2. Differentially expressed proteins in exosomes from liver metastases and non-metastasis CRC patients.**

| Accession | Name | Symbol |
| --- | --- | --- |
| sp\|O43854\|EDIL3_HUMAN | EGF-like repeat and discoidin I-like domain-containing protein 3 | EDIL3 |
| sp\|P05121\|PAI1_HUMAN | Plasminogen activator inhibitor 1 | SERPINE1 |
| sp\|P21926\|CD9_HUMAN | CD9 antigen | CD9 |
| sp\|P05026\|AT1B1_HUMAN | Sodium/potassium-transporting ATPase subunit beta-1 | ATP1B1 |
| sp\|P35625\|TIMP3_HUMAN | Metalloproteinase inhibitor 3 | TIMP3 |
| sp\|P07355\|ANXA2_HUMAN | Annexin A2 | ANXA2 |
| sp\|P62979\|RS27A_HUMAN | Ubiquitin-40S ribosomal protein S27a | RPS27A |
| sp\|Q8WUJ3\|CEMIP_HUMAN | Cell migration-inducing and hyaluronan-binding protein | KIAA1199 |
| sp\|P08758\|ANXA5_HUMAN | Annexin A5 | ANXA5 |
| sp\|P05556\|ITB1_HUMAN | Integrin beta-1 | ITGB1 |
| sp\|Q9NQW7 \|XPP1_HUMAN | Xaa-Pro aminopeptidase 1 | XPNPEP1 |
| sp\|P08195\|4F2_HUMAN | 4F2 cell-surface antigen heavy chain | SLC3A2 |
| sp\|P78536\|ADA17_HUMAN | Disintegrin and metalloproteinase domain-containing protein 17 | ADAM17 |
| sp\|P53990\|IST1_HUMAN | IST1 homolog | IST1 |
| sp\|P27348\|1433T_HUMAN | 14-3-3 protein theta | YWHAQ |
| sp\|P49641\|MA2A2_HUMAN | Alpha-mannosidase 2x | MAN2A2 |
| sp\|Q14204\|DYHC1_HUMAN | Cytoplasmic dynein 1 heavy chain 1 | DYNC1H1 |
| sp\|O95084\|PRS23_HUMAN | Serine protease 23 | PRSS23 |
| sp\|Q08722\|CD47_HUMAN | Leukocyte surface antigen CD47 | CD47 |
| sp\|P00750\|TPA_HUMAN | Tissue-type plasminogen activator | PLAT |
| sp\|Q07954 \|LRP1_HUMAN | Prolow-density lipoprotein receptor-related protein 1 | LRP1 |
| sp\|Q8WUJ3\|CEMIP_HUMAN | Cell migration-inducing and hyaluronan-binding protein | CEMIP |
| sp\|Q9UL62 \|TRPC5_HUMAN | Short transient receptor potential channel 5 | TRPC5 |
| sp\|P08962\|CD63_HUMAN | CD63 antigen | CD63 |

**
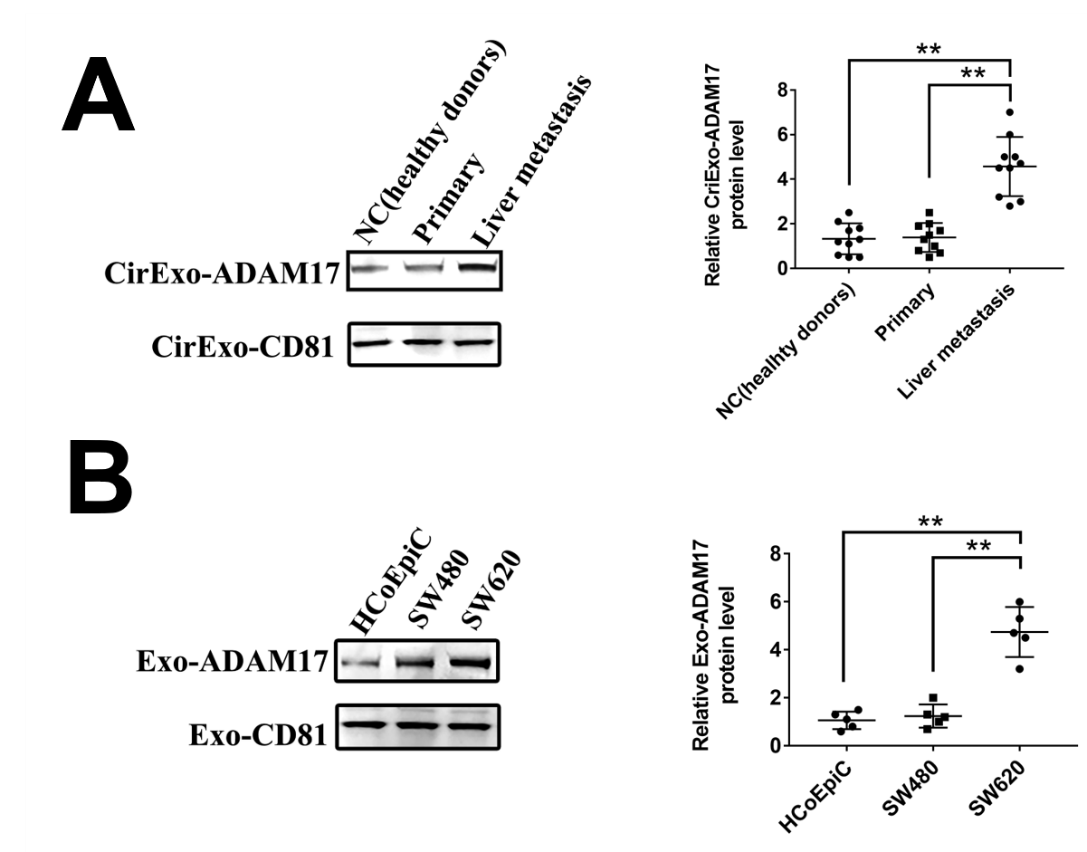
Figure S1**

**Figure S1 ADAM17 levels in exosomes from healthy donors’ sera and normal cell lines.** (A) The serum-derived exosomes of NC (healthy donors), and non-metastatic (primary) and liver metastatic patients with CRC were collected and lysed for analysis of ADAM17 protein levels. The relative protein levels were further calculated via gray analysis (the total exosomal protein was set as the internal control, n=10). (B) HCoEpiC, SW480, and SW620 exosomes were subjected to analysis of ADAM17 protein levels in conditioned medium. The relative protein levels were further calculated via gray analysis (the total exosomal protein was set as the internal control for exosomal protein analysis, n=6). Data are expressed as means ± SDs. *, P < 0.05; **, P < 0.01.

**
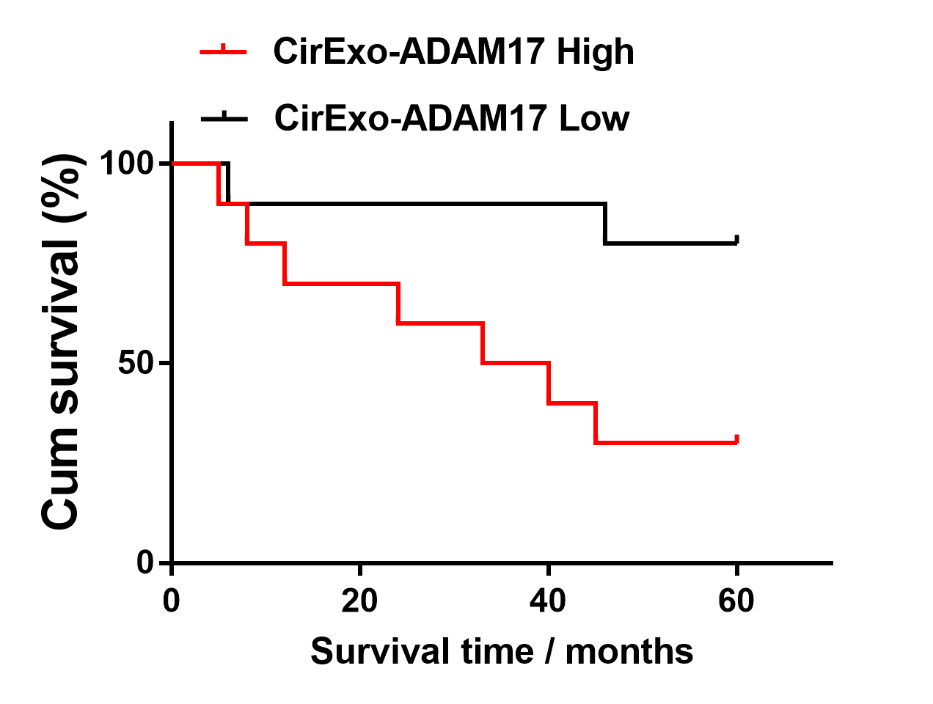
Figure S2**

**Figure S2 Overall survival curve comparison of colorectal cancer patients with different CirExo-ADAM17 level.**

**Table S3: Univariate analysis of prognostic factor-** **CirExo-ADAM17 in patients with colorectal cancer by Kaplan-Meier**

| CirExo-  ADAM17 | Cases, *n* | 3-year survival rate (%) | 5-year survival rate (%) | Average survival month | P value |
| --- | --- | --- | --- | --- | --- |
| High | 10 | 50% | 30% | 36.5 |  |
| Low | 10 | 90% | 80% | Undefined | 0.0291 |
